# FOXP4‐AS1 is a favorable prognostic-related enhancer RNA in ovarian cancer

**DOI:** 10.1042/BSR20204008

**Published:** 2021-04-30

**Authors:** Tian Hua, Yun-jie Tian, Rui-min Wang, Cai-fen Zhao, Yun-hong Kong, Rui-qing Tian, Wei Wang, Li-xia Ma

**Affiliations:** 1 Department of Gynaecology, Affiliated Xing Tai People Hospital of Hebei Medial University, 399 Shunde Road, Xingtai 054001, China; 2 Department of Obstetrics and Gynaecology, Hebei Medical University, Fourth Hospital, 12 Jiankang Road, Shijiazhuang 050000, China; 3 Department of Obstetrics and Gynaecology, Hebei Medical University, Second Hospital, Shijiazhuang 050000, China

**Keywords:** enhancer, FOXP4, FOXP4-AS1, OV, prognosis

## Abstract

Ovarian cancer (OV) is the main cause of deaths worldwide in female reproductive system malignancies. Enhancer RNAs (eRNAs) are derived from the transcription of enhancers and has attracted increasing attention in cancers recently. However, the biological functions and clinical significance of eRNAs in OV have not been well described presently. We used an integrated data analysis to identify prognostic-related eRNAs in OV. Tissue-specific enhancer-derived RNAs and their regulating genes were considered as putative eRNA–target pairs using the computational pipeline PreSTIGE. Gene expression profiles and clinical data of OV and 32 other cancer types were obtained from the UCSC Xena platform. Altogether, 71 eRNAs candidates showed significant correlation with overall survival (OS) of OV samples (Kaplan–Meier log-rank test, *P*<0.05). Among which, 23 were determined to be correlated with their potential target genes (Spearman’s r > 0.3, *P*<0.001). It was found that among the 23 prognostic-related eRNAs, the expression of forkhead box P4 antisense RNA 1 (FOXP4-AS1) had the highest positive correlation with its predicted target gene *FOXP4* (Spearman’s r = 0.61). Moreover, the results were further validated by RT-qPCR analysis in an independent OV cohort. Our results suggested the eRNA FOXP4-AS1 expression index may be a favorable independent prognostic biomarker candidate in OV.

## Introduction

Ovarian cancer (OV) is the fifth malignant reproductive tumor in women leading to mortality [1]. The Surveillance, Epidemiology and End Results (SEER) Program reported United States would have 21750 new diagnosed cases and 13940 new deaths in women would be seen in 2020 [2]. During the past 30 years, the survival in OV has barely improved though chemotherapy drugs and surgical approaches had obtained impressive advances, with 5-year survival remaining approx. 30% for patients in advanced stages [3]. Thus, it remains an urgent issue to explore underlying molecular mechanism for OV progression.

Enhancer RNA (eRNA) is considered to be a type of long noncoding RNAs (lncRNAs) transcribed from putative enhancer regions [4]. However, the functions of eRNAs remain enigmatic presently. It was suggested that enhancer transcription is the noisy byproduct of transcription machinery [5]. However, more and more studies have demonstrated the importance of eRNAs in transcriptional machinery to mediate the target genes transcription [6,7]. Moreover, some studies showed that knockdown of the eRNA was associated with the down-regulation of target genes [8–10]. In human cancers, the enhancer activation and production of eRNAs were highly associated with the dysregulation of tumor oncogenes, tumor suppressor genes and other stimuli [11,12]. Current models suggested that eRNAs interact with RNA polymerase II (RNA pol II), mediators and transcription factors to promote promoter–enhancer looping and the consequent up-regulation of the corresponding target genes [13]. Research by Li et al. [14] pointed out that widely estrogen-induced eRNA transcription was associated with up-regulated corresponding genes in hormone-dependent tumor breast cancer cells. Zhao et al. [15] found PSA eRNA can *cis* and *trans* regulate expression of a subclass of genes involved in androgen action and cancer progression in castration-resistant prostate cancer cells. Some eRNAs are related to clinical features of cancer or even patients survival [16]. Zhao et al. [17] identified strong correlation of eRNA expression and smoking history (SCRIBe), grade (APH1Ae), stage (CELF2e), subtype (EN1e) and survival (TAOK1e and NET1e). However, the biological functions and clinical significance of eRNAs in OV have not been well described presently.

Above all, our study aims to explore certain prognostic-related eRNAs in OV patients based on bioinformatics analysis. A total of 71 eRNAs were significantly correlated with overall survival (OS) of OV patients from The Cancer Genome Atlas (TCGA). Among these 71 eRNAs, 23 were determined to be correlated with their potential target genes. The functional lncRNA forkhead box P4 antisense RNA 1 (FOXP4-AS1) located within the tissue-specific enhancer and its nearby gene forkhead box P4 (*FOXP4*) showed the highest correlation using PreSTIGE algorithm and UCSC Xena browser (http://xenabrowser.net). Furthermore, we also validated the effect of FOXP4-AS1 on clinical outcome as an important prognostic-related eRNA in an independent OV cohort.

## Materials and methods

### Acquisition of eRNAs data

PreSTIGE was able to predict the enhancers through identifying protein-coding genes with increased tissue-specific expression. PreSTIGE was a method based on the assumption that these genes are targets of tissue-specific enhancers within the specified domain size [18]. In this current study, a slightly modified version of PreSTIGE was employed to address the association of lncRNA with enhancer function, in which CTCF domains are excluded with the domain size expanded to 200 kb across the transcription start sites of the protein-coding genes. In addition, all enhancers that were overlapping with their predicted targets were screened from the enhancer datasets used in the subsequent analysis [19]. A previous study showed that AP001056.1 is a key immune-related eRNA in squamous cell carcinoma of the head and neck, which was associated with clinical outcomes through the above-mentioned method [20].

Mapping between gene symbol and Ensembl transcript ID was obtained by Ensembl BioMart. UCSC Xena browser [21] was also3 used to collect clinical data of cancer and putative eRNAs’ levels. Candidate key eRNAs in OV were set as eRNAs with significant correlations with target genes levels (*P*<0.001, r > 0.3) and OS (*P*<0.05).

### Functional enrichment analysis

Gene Ontology (GO) functional analysis was performed using cluster Profiler package of R software, so as for Kyoto Encyclopedia of Genes and Genomes (KEGG) pathway analysis of eRNA-related coding genes based on co-expression screening.

### Survival and hazard analyses

In the present study, FOXP4-AS1 expressions were divided into FOXP4-AS1^high^ and FOXP4-AS1^low^ groups according to median expression value. Kaplan–Meier with log‐rank test, univariate and multivariate Cox regression analyses were used to compare prognostic differences between different FOXP4‐AS1 expression groups. Independent factors for survival were determined by Cox regression model. FOXP4-AS1 levels have also been investigated on its association with clinical characteristics, during which comparison between clinical variables in two groups was performed using Wilcoxon rank-sum test.

### Sampling of tumor specimens

Tissue samples were collected from 42 patients with histologically confirmed OV between May 2017 and May 2018. All patients received cytoreductive surgery debulking followed platinum-based chemotherapy and received no treatment prior to surgery. Tumor tissue specimens were stored in RNA later solution (Ambion, Carlsbad, CA, U.S.A.) immediately after surgical removal, incubated at 4°C for at least 24 h and subsequently stored at −20°C until needed.

The study was approved by the Ethics Committee of Affiliated Xing Tai People Hospital of Hebei Medial University Hospital (2020 [015]). All patients provided written informed consent.

### Quantification of FOXP4-AS1 and FOXP4 transcript levels in tissue samples

Total RNA was extracted using TRIzol reagent (Generay Biotech, Shanghai, Co., Ltd., China), according to manufacturer’s protocol. Revert-Aid First Strand cDNA Synthesis Kit (Thermo Scientific, U.S.A.) was used to synthesize cDNA from 500 ng total RNA. Transcript ID: ENSG00000234753.5 (for gene *FOXP4-AS1*) and ENSG00000137166.15 (for gene *FOXP4*). Reverse transcription quantitative PCR was done using QuantiNova TMSYBR® Green PCR Kit (Qiagen, Hilden, Germany). Custom primers for FOXP4-AS1 (forward: TCTCCAACTCCTCTGCTCCAATCC reverse: GCTCCGCTGCCTGTGACAAG) and FOXP4 (forward: GCCTGCTCTCTGCTCACAAGAAG reverse: GCCATCTCCTACCTGTCCCTCAC) were obtained from Sangon Biotech Co. Ltd. (Shanghai, China). GAPDH was used as housekeeping gene. Each sample was measured in triplicate.

### Statistical analysis

All statistical analyses were performed using R software (Version 3.6.3). Comparisons between two groups were performed using Wilcoxon rank-sum test and comparisons between three or more groups were done by using Kruskal–Wallis test. Survival analysis was done by Kaplan–Meier analysis. The independent factors were determined by univariate and multivariate Cox regression analyses. *P*-values <0.05 were set as statistically significant.

## Results

### Prognostic-related eRNAs screening in OV

Taking advantage of the PreSTIGE algorithm, 2695 lncRNA transcripts labeled by active tissue-specific enhancers derived ENCODE (Encyclopedia of DNA Elements database) and 2303 predicted target genes were identified. The clinical data of TCGA OV cohort came from UCSC Xena browser, as shown in Table 1. The conversion of transcript ID into gene ID were carried out by Ensembl BioMart. After that, 2695 transcripts were mapped to their corresponding 1288 genes. And eventually using Kaplan–Meier log-rank test, 71 gene were identified to be associated with OS significantly from these 1288 genes (Supplementary Table S1; Kaplan–Meier log-rank test, *P*<0.05). Spearman’s rank correlation results showed that significant correlations were observed in just 23 of these 71 eRNAs when comparing them with predicted target gene mRNA levels, as shown in Table 2 (coefficient r > 0.3, *P*<0.001).

**Table 1:** The clinical parameters in TCGA OV cohort

Characteristics		Percentage
**Age**	≤65	260 (68.6%)
	>65	119 (31.4%)
**Stage**	Stages I–II	24 (6.33%)
	Stages III–IV	352 (92.88%)
	Unknown	3 (0.79%)
**Lymphatic_invasion**	No	48 (12.66%)
	Unknown	230 (60.69%)
	Yes	101 (26.65%)
**Grade**	G1	1 (0.26%)
	G2	45 (11.87%)
	G3	322 (84.96%)
	Unknown	11 (2.9%)
**Person_neoplasm_cancer_status**	Tumor free	84 (22.16%)
	unknown	48 (12.66%)
	With tumor	247 (65.17%)
**Primary_therapy_outcome_success**	CR+PR	256 (67.55%)
	PD+SD	49 (12.92%)
	Unknown	74 (19.53%)
**Tumor_residual_disease**	>10 mm	97 (25.59%)
	1–10 mm	171 (45.12%)
	0 mm	67 (17.68%)
	unknown	44 (11.61%)

**Table 2:** List of OS-associated eRNAs derived from enhancers in OV

eRNAs	TANRIC OS analysis, log-rank *P*-value	Predicted target genes	Correlation between eRNA and the target	
			Correlation coefficient r	*P*-value
SLC44A3-AS1	0.046	*SLC44A3*	0.665	0
SLC2A1-AS1	0.037	*SLC2A1*	0.455	0
FLVCR1-DT	0.011	*FLVCR1*	0.401	0
AC141930.1	0.048	*PXDN*	0.482	1.71E-23
HAGLROS	0.013	*HOXD1*	0.619	1.52E-41
HAGLROS	0.013	*HOXD3*	0.607	0
HAGLROS	0.013	*HOXD4*	0.599	2.63E-38
HAGLROS	0.013	*HOXD8*	0.502	0
LNC-LBCS	0.032	*ID4*	0.481	2.00E-23
HCP5	0.036	*MICB*	0.502	0
HCP5	0.036	*HCP5*	1	0
FOXP4-AS1	0.005	*FOXP4*	0.609	0
HIVEP2	0.043	*HIVEP2*	1	0
Z94721.1	0.048	*FGFR1OP*	0.568	6.90E-34
LINC00996	0.013	*GIMAP4*	0.573	1.39E-34
LINC00996	0.013	*GIMAP6*	0.550	2.20E-31
LINC00996	0.013	*GIMAP7*	0.579	2.17E-35
LINC00996	0.013	*GIMAP8*	0.534	2.42E-29
MAL2	0.006	*MAL2*	1	0
ZFHX4-AS1	0.021	*ZFHX4*	0.648	1.19E-46
AC067930.2	0.006	*MAPK15*	0.429	1.93E-18
GAS1RR	0.045	*GAS1*	0.625	0
CALML3-AS1	0.034	*NET1*	0.418	1.69E-17
AP002754.1	0.022	*SYT7*	0.620	1.14E-41
PAAF1	0.024	*PAAF1*	1	0
AC007848.1	0.048	*NTF3*	0.430	1.47E-18
ITFG2-AS1	0.021	*FKBP4*	0.407	0
IFNG-AS1	0.011	*IFNG*	0.519	1.30E-27
MRPS31P5	0.008	*ATP7B*	0.481	2.32E-23
MRPS31P5	0.008	*NEK3*	0.487	5.53E-24
LINC00665	0.011	*ZFP14*	0.541	0
LINC00665	0.011	*ZFP82*	0.666	0
LINC00665	0.011964	*ZNF146*	0.539671	4.98E-30
LINC00665	0.011964	*ZNF260*	0.583943	0

### eRNA FOXP4-AS1 showed the highest positive correlation with its target gene FOXP4 in OV

**Figure 1:**
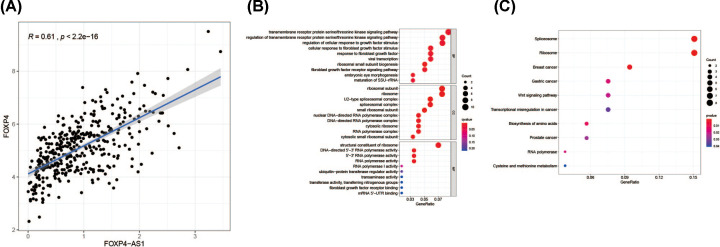
The correlation between FOXP4‐AS1 and FOXP4 expression in OV (**A**) Scatterplot showing the association between FOXP4‐AS1 and FOXP4 levels according to TANRIC (the Atlas of Noncoding RNAs in Cancer) correlation analysis. (**B,C**) Significant GO and KEGG pathway analysis of the FOXP4‐AS1 co‐expression genes.

Among the 23 prognostic-related eRNAs, it was found that the expression of FOXP4-AS1 has the highest positive correlation with its predicted target gene *FOXP4* (Figure 1A; Spearman’s r = 0.61, *P*<0.001). Therefore, FOXP4-AS1 was confirmed as the research object from the 23 eRNAs in the present study. Another 185 co‐expression genes also showed significant correlation with FOXP4-AS1 (Supplementary Table S3; r > 0.3, *P*<0.001). To analyze pathways and biological functions, KEGG pathway and GO term analyses were performed on FOXP4-AS1 co‐expression genes (Figure 1B,C). The significantly enriched GO terms included transmembrane receptor protein serine/threonine kinase signaling pathway, RNA polymerase activity and ribosomal subunit. KEGG pathway analysis presented spliceosome, breast cancer, transcriptional misregulation in cancer and Wnt signaling pathway were also enriched according to KEGG pathway analysis.

### High expression of FOXP4‐AS1 correlates with the favorable prognosis of OV patients

**Figure 2:**
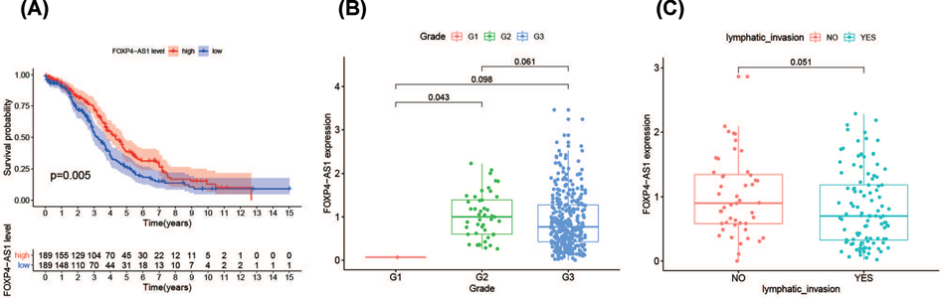
The impact of eRNA FOXP4‐AS1 on the prognosis of OV (**A**) Kaplan–Meier OS curve for OV patients with FOXP4‐AS1^high^ and FOXP4‐AS1^low^ expression, obtained through the TANRIC platform. (**B,C**) Clinical relevance of eRNA FOXP4‐AS1 expression in OV patients.

The patients were divided into low and high groups based on the median value of FOXP4-AS1 expression in TCGA cohort. The FOXP4-AS1^high^ group showed longer OS when compared with FOXP4-AS1^low^ group (*P*=0.005; Figure 2A). Multivariable analysis of survival showed the FOXP4-AS1 expression levels were significantly associated with OS when tumor residual size, grade, stage and age were included (*P*=0.023, HR = 0.741, 95% CI = 0.572–0.959, Table 3). Moreover, lower FOXP4-AS1 was more associated with higher grade and lymphatic invasion compared with higher FOXP4-AS1 (Figure 2B,C). These results showed that for OV patients FOXP4-AS1 may be a favorable independent prognostic biomarker candidate.

**Table 3:** Prognostic factors in TCGA OV cohort using the Cox proportional hazards model

	OS		
	HR	95% CI	*P*
**Age**	1.019	1.004–1.034	0.008
≤65 vs >65			
**FIGO stage**	1.094	0.783–1.528	0.596
I–II vs III–IV			
**Grade**	1.381	0.886–2.152	0.153
G1–2 vs G3			
**Person_neoplasm_cancer_status**	9.108	4.420–18.765	2.1E-09
Tumor vs tumor-free			
**Tumor residual size**	1.148	0.905–1.456	0.254
0 mm vs ≤0–10 mm vs >10 mm			
**FOXP4-AS1 expression**	0.741	0.572–0.959	0.023
High vs low			

### Validation of the role of FOXP4‐AS1 in an independent OV cohort

**Figure 3:**
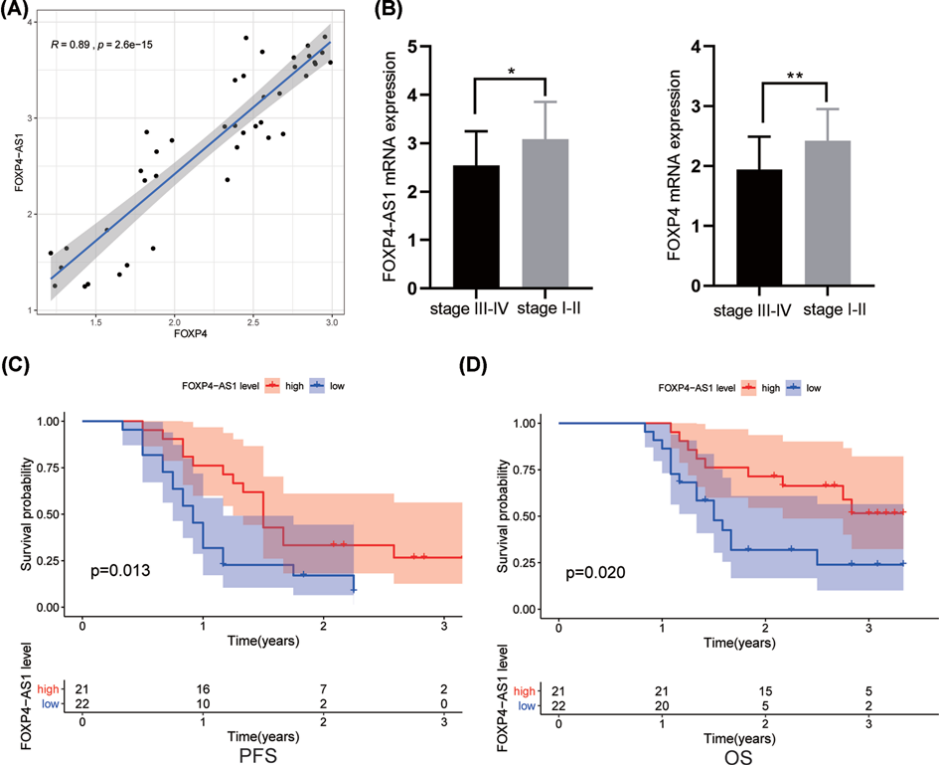
Validation of the role of FOXP4‐AS1 in an independent OV cohort (**A**) Scatterplot showing correlation between FOXP4-AS1 and FOXP4 mRNA in OV tumor samples. (**B**), The comparison of FOXP4-AS1 and FOXP4 between early stage patients and advanced stage patients. (**C,D**) Kaplan–Meier survival curve for OV patients with FOXP4‐AS1^high^ and FOXP4‐AS1^low^ expression. *P<0.05, **P<0.01.

Archived information of 42 OV patients was obtained from the Affiliated Xing Tai People Hospital of Hebei Medial University. All patients received platinum-based chemotherapy following primary debulking surgery and were followed up for 3 years. The median age was 55.2 years (ages ranged from 36 to 71). Among them, 42 patients were histologically diagnosed with high-grade ovarian serous carcinoma. According to FIGO (International Federation of Gynecology and Obstetrics) staging, 29 (69.04%) and 13(30.96%) cases had stage III–IV and I–II OV, respectively. The characteristics of recruited subjects are listed in Supplementary Table S2. The expression of FOXP4-AS1 and FOXP4 mRNA levels were tested in the 42 tumor samples. Spearman’s rank correlation showed that a significantly positive correlation exists between FOXP4-AS1 and FOXP4 (r = 0.89, *P*<0.001, Figure 3A). The expressions of FOXP4-AS1 and FOXP4 in stage III–IV were both lower than those in stages I–II OV (*P=0.023, P=0.009*, Figure 3B). The 42 patients were divided into low and high groups based on the median value of FOXP4-AS1 expression. Kaplan–Meier analysis indicated that the progression-free survival (PFS) and OS of patients with FOXP4-AS1^low^ group were shorter compared with FOXP4-AS1^high^ group (Figure 3C,D).

### Pan-cancer analysis of FOXP4-AS1

**Figure 4:**
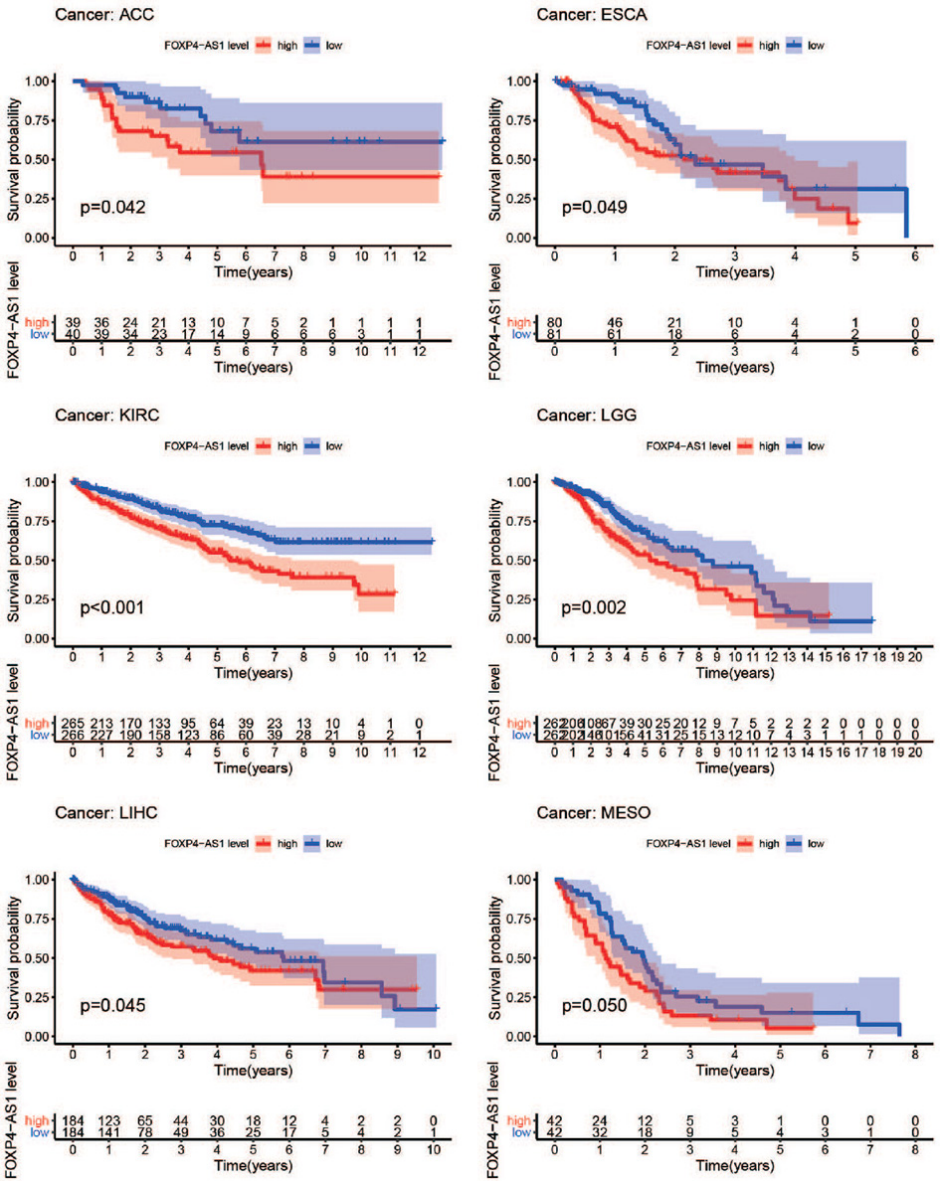
The prognostic effect of FOXP4-AS1 in other TCGA cancer types was investigated using UCSC Xena browser

To evaluate tissue-specificity of FOXP4-AS1, we further investigated its role in pan-cancer using UCSC Xena browser (http://xenabrowser.net). As summarized in Table 4, the significant correlations between FOXP4-AS1 and FOXP4 existed in the 27 tumor types. Moreover, the prognostic effect of FOXP4-AS1 in pan-cancer was also explored. Besides OV, the significant impact of FOXP4-AS1 on survival was also observed in Adrenocortical carcinoma (ACC), Esophageal carcinoma (ESCA), Kidney renal clear cell carcinoma (KIRC), Lower Grade Glioma (LGG), Liver hepatocellular carcinoma (LIHC) and Mesothelioma (MESO) (*P*<0.05, Figure 4).

**Table 4:** The correlations for *FOXP4-AS1* and *FOXP4* across 33 cancer types from TCGA project

eRNA	Target	Cancer type	Details	Correlation coefficient r	Correlation *P*-value
FOXP4-AS1	*FOXP4*	ACC	Adrenocortical carcinoma	0.379016	0.000572
FOXP4-AS1	*FOXP4*	BLCA	Bladder Urothelial Carcinoma	0.412087	2.80E-18
FOXP4-AS1	*FOXP4*	BRCA	Breast invasive carcinoma	0.545097	0
FOXP4-AS1	*FOXP4*	CESC	Cervical squamous cell carcinoma and endocervical adenocarcinoma	0.532577	8.13E-24
FOXP4-AS1	*FOXP4*	CHOL	Cholangiocarcinoma	0.553153	0.000574
FOXP4-AS1	*FOXP4*	COAD	Colon adenocarcinoma	0.332022	1.88E-13
FOXP4-AS1	*FOXP4*	DLBC	Lymphoid neoplasm diffuse large B-cell lymphoma	0.630265	2.83E-06
FOXP4-AS1	*FOXP4*	ESCA	Esophageal carcinoma	0.768508	0
FOXP4-AS1	*FOXP4*	GBM	Glioblastoma multiforme	0.102472	0.186249
FOXP4-AS1	*FOXP4*	HNSC	Head and neck squamous cell carcinoma	0.480517	2.31E-30
FOXP4-AS1	*FOXP4*	KICH	Kidney chromophobe	0.348383	0.004659
FOXP4-AS1	*FOXP4*	KIRC	Kidney renal clear cell carcinoma	0.378652	1.11E-19
FOXP4-AS1	*FOXP4*	KIRP	Kidney renal papillary cell carcinoma	0.276898	1.92E-06
FOXP4-AS1	*FOXP4*	LAML	Acute myeloid leukemia	0.65727	4.92E-20
FOXP4-AS1	*FOXP4*	LGG	Lower grade glioma	0.287832	1.50E-11
FOXP4-AS1	*FOXP4*	LIHC	Liver hepatocellular carcinoma	0.517671	5.01E-27
FOXP4-AS1	*FOXP4*	LUAD	Lung adenocarcinoma	0.476887	3.18E-31
FOXP4-AS1	*FOXP4*	LUSC	Lung squamous cell carcinoma	0.434769	1.62E-24
FOXP4-AS1	*FOXP4*	MESO	Mesothelioma	0.773174	2.71E-18
FOXP4-AS1	*FOXP4*	OV	Ovarian serous cystadenocarcinoma	0.60923	0
FOXP4-AS1	*FOXP4*	PAAD	Pancreatic adenocarcinoma	0.556598	0
FOXP4-AS1	*FOXP4*	PCPG	Pheochromocytoma and paraganglioma	0.529036	1.37E-14
FOXP4-AS1	*FOXP4*	PRAD	Prostate adenocarcinoma	0.003991	0.929118
FOXP4-AS1	*FOXP4*	READ	Rectum adenocarcinoma	0.185807	0.016324
FOXP4-AS1	*FOXP4*	SARC	Sarcoma	0.713188	3.69E-42
FOXP4-AS1	*FOXP4*	SKCM	Skin cutaneous melanoma	0.510865	1.13E-32
FOXP4-AS1	*FOXP4*	STAD	Stomach adenocarcinoma	0.537309	0
FOXP4-AS1	*FOXP4*	TGCT	Testicular germ cell tumors	0.894347	1.14E-55
FOXP4-AS1	*FOXP4*	THCA	Thyroid carcinoma	0.473497	0
FOXP4-AS1	*FOXP4*	THYM	Thymoma	0.535237	4.59E-10
FOXP4-AS1	*FOXP4*	UCEC	Uterine corpus endometrial carcinoma	0.282166	1.73E-11
FOXP4-AS1	*FOXP4*	UCS	Uterine carcinosarcoma	0.593096	2.41E-06
FOXP4-AS1	*FOXP4*	UVM	Uveal melanoma	0.306446	0.005879

## Discussion

eRNAs have attracted particular interest due to their potential roles in mediating enhancer functions and gene transcription associated with cancers [22]. The aim of the present study is to explore the eRNAs as prognostic related biomarker in OV. The results revealed that: (1) for the first time, FOXP4-AS1 was determined to be an eRNA with top positive correlation with its target gene *FOXP4* among the prognosis-related eRNAs in OV. (2) FOXP4-AS1 may be an independent biomarker in OV patients‘ prognosis. (3) The prognostic significance of FOXP4-AS1 as well as the positive correlation with FOXP4 were further validated in an independent OV cohort. (4) The impact of FOXP4-AS1 on survival was presented in pan-cancer analysis.

**Figure 5:**
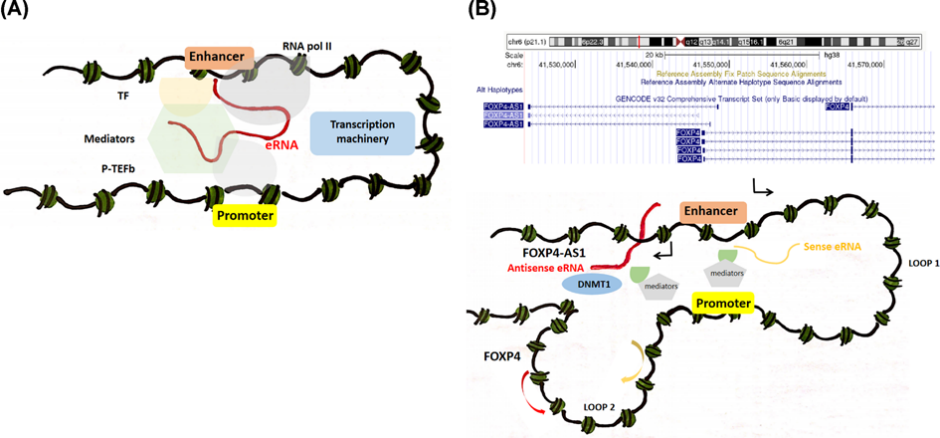
The activation of genes near enhancer (**A**) The model of the activation of genes near enhancer through the inhibition of RNAPII release. (**B**) The mechanism of antisense eRNA activating mRNA, a possible mechanism of how FOXP4-AS1 contributes to FOXP4 transcription.

To date, several models have been developed to show how eRNAs regulate the transcription of targeted genes. However, the mechanisms are quite different [23]. In addition to promoter–enhancer looping and histone modifications, eRNAs promote targets transcription through enhancing the binding of RNA Pol II or leasing negative regulator [22]. As shown in Figure 5A, eRNAs can independently recruit RNA Pol II to both enhancer and promoter loci. Once recruited, eRNAs interact with the NELF and P-TEFb complexes to regulate pause-release of RNA Pol II and promote enhancer and gene transcription [24]. However, compared with sense eRNAs, the mechanism of antisense eRNAs in regulation of target genes was kind of different. Pan et al. [25] found that antisense eRNA mediated mRNA through binding to DNMT1. Promoter–gene-ending interaction increases the specific antisense eRNA targeting gene-ending region. In the present study, FOXP4-AS1 was found to be top correlated with its predicted target FOXP4 in OV. Furthermore, the correlation was validated in an independent 42 OV patients. Similarly, Li et al. [26] reported that FOXP4-AS1 and FOXP4 were up-regulated in esophageal carcinoma samples and were positively correlated with each other based on bioinformatics prediction. Therefore, we speculated that the regulation of antisense eRNA in the predicted targets may be a possible mechanism of how FOXP4-AS1 contributes to FOXP4 transcription (Figure 5B). Our study presented the putative regulatory roles of eRNA FOXP4-AS1 on target gene FOXP4 in OV, nevertheless, further experiments are still necessary to confirm the underlying mechanism.

FOXP4-AS1 is an emerging cancer-related biomarker, whose roles in different types of cancers were inconsistent. As previous research reported, FOXP4-AS1 was confirmed to be an oncogene in colorectal cancer associated with a dismal prognosis [27]. The consistent results were also shown in prostate cancer [28], pancreatic ductal adenocarcinoma [29], and osteosarcoma [30]. More recently, Binang et al. pointed out that increased FOXP4-AS1 expression was associated with beneficial outcomes considering gastric cancer disease-free survival based on public databases [31]. In this study, high FOXP4-AS1 expression was related to better OS based on TCGA OV patients. Multivariable analysis of survival showed the FOXP4-AS1 expression levels were significantly associated with OS when tumor residual size, grade, stage, and age were included. Additionally, FOXP4-AS1 expression showed a correlation with some cancer-related clinical features including clinical stage, grades and lymphatic invasion. In the validation stage, the FOXP4-AS1 high group is closely associated with better survival than the FOXP4-AS1 low group in a single OV cohort. In summary, FOXP4-AS1 may play a role of tumor suppressor in OV. However, strong correlations between the high expression of FOXP4-AS1 and poor prognosis were found in pan-cancer survival analysis including ACC, ESCA, LIHC, brain LGG, KIRC, MESO. Compared with these types of cancer, the different prognosis values of FOXP4-AS1 in OV may be attributed to the cancer type-specific eRNAs. Approximately 59% of all identified eRNAs (5332 out of 9108) were confirmed to be cancer type-specific from a previous report [17], from which we know cancer type and unique pattern were closely associated for eRNA expression. Thus, FOXP4-AS1 expression pattern may be obviously diverse in the different cancer cells. Our results supplied more information on the potential prognosis-related role of FOXP4-AS1 in different cancer types. The converse impact of FOXP4-AS1 on survival in OV made it a noteworthy eRNA for this tumor type.

FOXP4, one member of the forkhead box P family [32], was shown to be most positively correlated with FOXP4-AS1 expression among the 186 potential target genes depending our criteria in this study. Moreover, positive correlations of the two genes were also observed in the pan-cancer analysis. FOXP4 was observed in different cancer types as a tumor suppressor or oncogene previously [33]. FOXP4 positively regulated by eRNA FOXP4-AS1 may be one of the mechanisms that involved in the biological processes of OV. In addition, the other potential target genes and transcripts form the functional clusters may be majorly involved in cancer progression in OV. It cannot be ignored the influence through sponging miRNAs, lncRNAs regulate mRNA expression of these targets as ceRNAs on post-transcriptional level. The functional role of eRNA FOXP4-AS1 in OV remains need to determine *in vivo* and *in vitro* experiments.

## Conclusion

In this current study, we explored the prognosis-related eRNAs in OV patients as well as related target genes. Study results suggested FOXP4-AS1 may be a favorable independent prognostic biomarker candidate in OV with top positive correlation with its target gene *FOXP4*. However, the role of FOXP4-AS1 in prognosis was inconsistent in pan-cancer analysis. Moreover, the target genes of FOXP4-AS1 are mainly involved in cancers, implying the importance of eRNA FOXP4-AS1 in the biological progresses. These results provided new ideas in exploring the regulatory network of eRNAs in the process of OV evolution and prognosis, and thus to reveal the panorama of the disease. It should be a valuable field for further investigation of cancer therapy.

## Supplementary Material

Supplementary Tables S1-S3

## Data Availability

All datasets generated for the present study are included in the Supplementary material.

## Abbreviations

ACC: adrenocortical carcinoma
CTCF: CCCTC-Binding Factor
eRNA: enhancer RNA
ESCA: esophageal carcinoma
FOXP4: forkhead box P4
FOXP4-AS1: forkhead box P4 antisense RNA 1
GO: Gene Ontology
KEGG: Kyoto Encyclopedia of Genes and Genomes
KIRC: kidney renal clear cell carcinoma
LGG: lower grade glioma
LIHC: liver hepatocellular carcinoma
lncRNA: long noncoding RNA
MESO: mesothelioma
NELF: negative elongation factor
OS: overall survival
OV: ovarian cancer
P-TEFb: Positive transcription elongation factor b
RNA Pol II: RNA polymerase II
TCGA: The Cancer Genome Atlas
